# Resident perspectives on the value of interdisciplinary conference calls for geriatric patients

**DOI:** 10.1186/s12909-021-02750-4

**Published:** 2021-06-03

**Authors:** Roxana Naderi, Tyson A. Oberndorfer, Sarah R. Jordan, Blythe Dollar, Ethan U. Cumbler, Christine D. Jones

**Affiliations:** 1 Division of Hospital Medicine, Department of Medicine, University of Colorado Anschutz Medical Campus, 12401 E 17th Avenue, Mailstop F782, Aurora, CO 80045 USA; 2 Division of Geriatric Medicine, Department of Medicine, University of Colorado Anschutz Medical Campus, 12631 E 17th Avenue, Mailstop 8111, Aurora, CO 80045 USA; 3 Department of Medicine, University of Colorado Anschutz Medical Campus, 12631 E 17th Avenue, Aurora, CO 80045 USA; 4 Veterans Health Administration, Eastern Colorado Health Care System, Denver-Seattle Center of Innovation for Veteran-Centered and Value Driven Care, 1700 North Wheeling Street, Aurora, CO 80045-7211 USA

**Keywords:** Qualitative, Transitions of care, Resident education, Internal medicine, Geriatric medicine

## Abstract

**Background:**

There are limited competency-based educational curricula for transitions of care education (TOC) for internal medicine (IM) residency programs. The University of Colorado implemented a virtual interdisciplinary conference call, TEAM (Transitions Expectation and Management), between providers on the inpatient Acute Care of the Elder (ACE) unit and the outpatient Seniors Clinic at the University of Colorado Hospital. Residents rotating on the ACE unit participated in weekly conferences discussing Seniors Clinic patients recently discharged, or currently hospitalized, to address clinical concerns pertaining to TOC. Our goals were to understand resident perceptions of the educational value of these conferences, and to determine if these experiences changed attitudes or practice related to care transitions.

**Methods:**

We performed an Institutional Review Board-approved qualitative study of IM housestaff who rotated on the ACE unit during 2018–2019. Semi-structured interviews were conducted to understand perceptions of the value of TEAM calls for residents’ own practice and the impact on patient care. Data was analyzed inductively, guided by thematic analysis.

**Results:**

Of the 32 IM residents and interns who rotated on ACE and were invited to participate, 11 agreed to an interview. Three key themes emerged from interviews that highlighted residents’ experiences identifying and navigating some of their educational ‘blind spots:’ 1) Awareness of patient social complexities, 2) Bridging gaps in communication across healthcare settings, 3) Recognizing the value of other disciplines during transitions.

**Conclusions:**

This study highlights learner perspectives of the benefit of interdisciplinary conference calls between inpatient and outpatient providers to enhance transitions of care, which provide meaningful feedback and serve as a vehicle for residents to recognize the impact of their care decisions in the broader spectrum of patients’ experience during hospital discharge. Educators can maximize the value of these experiences by promoting reflective debriefs with residents and bringing to light previously unrecognized knowledge gaps around hospital discharge.

**Supplementary Information:**

The online version contains supplementary material available at 10.1186/s12909-021-02750-4.

## Background

In an era of rising costs of health care, increasing attention is being placed on improving the quality of care at hospital discharge in order to avoid readmissions and other adverse patient outcomes. Multiple components contribute to an ideal transition of care (TOC) following hospitalization, including communication of information, medication safety, optimization of community resources, patient education, care coordination across team members, and monitoring/managing symptoms after discharge [1]. Over the past several years, multiple programs have been developed and evaluated to improve transitions of care for older adults [2, 3]. In one recent study, the Extension for Community Health Outcomes-Care Transitions (ECHO-CT), a novel program involving interdisciplinary videoconferences for transitions of care was implemented for providers to discuss post-hospitalization skilled nursing facility (SNF) patients. This program led to a significant improvement in 30-day hospital readmissions, SNF length of stay, and 30-day total health care costs for patients [4]. However, models of integrated communication across sites of care have not been widely adopted, and many academic centers rely on written materials alone to bridge the gap between sites of care.

In 2012, the Alliance for Academic Internal Medicine (AAIM) Education Redesign Subcommittee proposed Entrustable Professional Activities (EPAs) for residency programs to achieve competency-based educational goals for residents, mandated by the Accreditation Committee of Graduate Medical Education (ACGME) [5, 6]. Despite EPA’s inclusion of educational goals like transitions of care and leading and working within an interprofessional health care team, few models exist to guide post-graduate curricula in these areas.

**Fig. 1:**
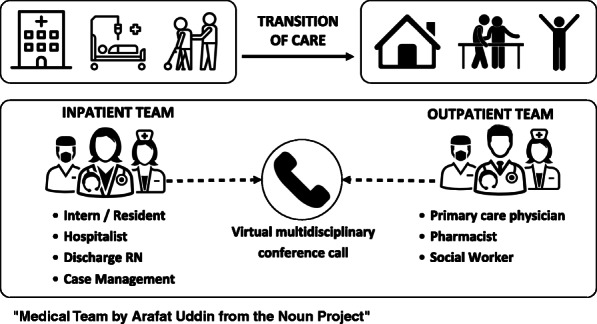
Transitions Expectation and Management (TEAM)  call visual model

Prior transitions of care curricula have been studied for their effect on clinical and patient-centered outcomes, but few studies have sought to better understand resident perceptions of these programs [7, 8]. In 2018, we implemented and adapted the ECHO-CT model to include weekly virtual interdisciplinary conference calls, referred to hereafter as Transitions Expectation and Management (TEAM) calls, between interprofessional providers on the geriatric inpatient unit of the University of Colorado Hospital and the Seniors Clinic (See Fig. 1). The goal of TEAM calls was to improve coordination around care transitions and to improve resident transitional care education. We completed a qualitative descriptive study to understand the perceived educational value of the interdisciplinary TEAM calls from the perspective of internal medicine interns and residents.

## Methods

### Design and setting

This is a qualitative descriptive study of resident experiences with weekly TEAM meetings on the Acute Care of the Elder (ACE) inpatient unit of an urban quaternary care academic medical center. As part of clinical rotations, internal medicine residents rotate on geriatric general medicine ward teams on the 21-bed ACE Unit, which specializes in the care of patients over 72 years of age. In September 2018, ACE unit leadership implemented new interdisciplinary weekly virtual TEAM calls between members of the inpatient ACE clinical service along with interprofessional staff of the outpatient geriatrics clinic (UCHealth Seniors Clinic) to discuss currently admitted and recently discharged mutual patients. Inpatient ACE team representatives on the calls included residents, the attending physician from the inpatient team, and the transitions of care nurse. Seniors Clinic representatives included members of the primary care provider team, social worker, nurses, and outpatient pharmacists. TEAM calls provided opportunities to clarify questions on medical care, identify risks to effective transition planning, and provide bi-directional feedback on effective execution of the intended plan and patient status following a transition of care. TEAM calls were facilitated using secure video conferencing to allow for real-time collaborative review of patient charts in the electronic medical record between both ACE and Seniors Clinic participants.

### Participants

Semi-structured interviews were completed with University of Colorado internal medicine residents rotating on the ACE unit who participated in TEAM calls between September 2018 and May 2019. Residents who rotated on ACE during this study period included those pursuing categorical internal medicine residency, customized internal medicine primary care or hospitalist training tracks, as well as preliminary medicine internship for specialties.

### Data collection

A convenience sampling strategy was employed as part of a larger program evaluation to determine the educational value of TEAM calls and understand learner perspectives on optimizing transitions of care curricula. All 32 internal medicine residents who participated in ACE-Seniors clinic conference calls between September 2018 and May 2019 were invited by email to participate in interviews [9, 10]. Up to three total emails were sent to residents to invite participation. A semi-structured interview guide (see Additional file 1) was used and included topics related to residents’ perceptions about the educational value of the TEAM calls to their own career trajectory, perceived time commitment, and impact on patient care from the calls. Individual interviews lasted up to 30 min and were completed either in person or over the telephone, per resident preference. The interviewer (BD) had prior experience conducting qualitative interviews and had no direct supervisory role for residents. Informed consent was completed prior to interviews, and participants were compensated $25 for interview completion. Interviews were audio recorded, professionally transcribed, and de-identified. This study was reviewed and deemed exempt by the Colorado Multiple Institutional Review Board.

### Data analysis

The team (SJ, CJ, RN, TO) used an inductive approach to first read a small set of transcripts individually and design a code book together, which was refined over time as the team met and iteratively reviewed codes to establish consensus. Each of the interview transcripts was then coded in Atlas.ti (Version 7, Berlin, Germany) by at least two coders using open coding guided by thematic analysis, and emerging themes were identified [11, 12]. A record of all analytical decisions and team discussions was maintained. Triangulation was conducted with peers practicing in hospital medicine (EC, CJ, RN) and geriatric medicine (TO) to enhance reliability [13].

## Results

We conducted 11 interviews with internal medicine residents who rotated on the ACE unit during 2018–2019. Of the residents interviewed, two were first-year intern residents, seven were in their second year, and two were in their third year. Four participants identified as female (36%). Seven interviewees were part of the Hospitalist Training Program within the residency, three were in the categorical track, and one was a preliminary resident.

Residents largely related their experiences on TEAM calls to their shared experience navigating educational ‘blind spots’ that they became aware of during their residency. These areas were commonly indicated by residents describing instances where “I didn’t know that” and “I never knew …” Interviews reflected a growing sense of awareness of the meaning and purpose underlying aspects of the work of managing transitions for complex vulnerable patients. One third-year resident explained how their attitude toward participating in the ACE-Seniors calls had evolved over time:

Initially it was a little like one extra thing they’re making us do, but as the weeks went on, I recognized that it was an important part in transitions of care and is something that I feel strongly can be a source of problems for patients when things get missed.

Three key themes emerged that further demonstrate awareness of educational value of the calls and residents’ ability to identify and reconcile these newfound ‘blind spots’ in their training and education: gaining awareness of patient social complexities, bridging gaps in communication across different health care settings, and recognizing the value of other disciplines during transitions. Data relating to these themes are represented in Table 1.

**Table 1:** Themes and illustrative quotations

Theme	Quotation
Awareness of patient social complexities	People still have issues going outside the hospital and people have chronic problems, and their hospital stay is just one event in their overall course. *#8 second year resident*
	The whole rotation in general cemented in my mind how difficult transitions of care are and how risky they are especially in the older population. Especially if there’s any sort of social circumstances, whether it be food scarcity or poor social support at home, or desire to stay independent versus continuing what our recommendations would be for safety... *#7 first year resident*
	At the end of the day, sometimes it’s all about, ‘Well no one can take out my dog when I’m in the hospital so I don’t want my appointment.’ So it’s more learning about our patients in accordance of them being sick and in accordance of them being human beings who live in society. They have things to do, bills to pay … so my job was to provide that information. *#3 second year resident*
Bridging gaps across healthcare settings	The idea of a hospitalist has been very much, ‘we’ll take care of them in the acute setting and then we’ll wash [our] hands of them,’ and getting that outpatient perspective- what’s actually happening with this patient- was helpful in terms of informing the way that I thought about other patient encounters. *#2 second year resident*
	There’s a lot of different people working with the patient, and it takes a lot of different people making a lot of different effort to make sure that everything goes smoothly- you need to keep that in mind when they’re discharged, so I think that was a helpful thing to learn and then reinforce in my everyday process. *#9 first year resident*
	It’s easy to just move on after you’ve discharged a patient, your part is done, but I do think it’s useful to be reminded that these transitions are real people on the other end getting the documentation. What you do echoes in time for that person over weeks or months or maybe their lifetime. *#5 second year resident*
	There are ways that we can have direct communication too, rather than just communicating through notes- as a hospitalist next year, it’s something that I’ll address with the community program that I’m working for- can we have a conference call for high risk patients and identify those? I think I can try to incorporate something very similar where the outpatient providers identify a high risk of re-admitting the patient and work on getting in touch with the hospitalist … see if I can implement that into my future practice. *#1 third year resident*
Recognizing the value of other disciplines across transitions	I didn’t know that pharmacy and social work were so intertwined in the post discharge care, so I thought that was really helpful that there was a chance to hand off to those people as well in addition to just their provider. *#11 second year resident*
	The case managers included in the call … they’re really the ones who are specializing in care transitions, and are especially knowledgeable about geriatric patient transitions. *#1 third year resident*
	Going over her [patient] medicines after discharge was so helpful because it was like, what do we need to make sure this patient really understands what’s happening and what were the things that led to her bounce backs the first time? Being able to have everybody in the same place to be able to talk about those things was helpful. *#2 second year resident*
	It was really useful having the pharmacist input to say, ‘this medication was actually put in incorrectly,’ and it was nice to know that someone else was looking at this and that things that really could have been near misses didn’t fall through the cracks. *#2 second year resident*

### Theme 1: Awareness of patient social complexities

Residents shared insights into socioeconomic complexities uncovered during TEAM conferences. They commented on how barriers to care became increasingly apparent from communication across disciplines and healthcare settings, including patients’ ability to afford medication, stability of patients’ home lives, and high risk of readmission with inadequate resources upon discharge. One first-year resident explained:

[ACE] puts you into a position where you’re not just thinking of the patient’s survival needs for a few days. You really have to try to see bigger — their overall care. Being more responsible for discharge … your job isn’t just to get them out of the hospital but to make sure that when they are discharged, they have the correct follow up, they can afford the prescriptions in the plan that you’re discharging with, the medications can be afforded and continued …

Another first-year resident demonstrated new appreciation of the importance of effective transitions of care planning, especially in older patients:

The whole rotation cemented in my mind how difficult transitions of care are and how risky they are, especially in the older population.

A second-year resident further expressed how TEAM calls prompted them to proactively identify potential causes for readmission and address those prior to discharge:

It was really good to see in a more real-world setting how their follow up takes place outside of the hospital — understanding the gaps people fall into and understanding appropriate ways to communicate to try and avoid those for patients across the board.

### Theme 2: Bridging gaps in communication across healthcare settings

Residents shared how TEAM calls reframed their perspectives on patients’ disparate experiences throughout different healthcare settings, helping them better contextualize their role within the larger order of patients’ movements through the healthcare system. A second-year resident specifically described a shift toward a more team-based approach between inpatient and outpatient providers:

You realize how much of an opportunity there is for you to be able to help other physicians, it’s just a reminder that my role is part of a continuum of care, and I need to be thinking about what ways I can contribute to that continuum and how I’m setting my colleagues up for success on the outpatient setting.

Another second-year resident felt that the direct communication with outpatient providers through TEAM calls also proved a valuable form of feedback beneficial for their own future practice:

Getting that outpatient perspective, what’s actually happening with this patient, was helpful in terms of informing the way that I thought about other patient encounters.

Residents recognized how TEAM calls framed their own perceptions of their position amidst transitions of care and reiterated their responsibilities and ability to improve transitions.

### Theme 3: Recognizing the value of other disciplines during transitions

Residents reflected on the value of interdisciplinary care during transitions. Acknowledging the importance of pharmacy involvement was common, with many trainees appreciating an added layer of oversight to reduce medication errors after discharge. Residents also noted the importance of social work and case management in facilitating “the care of the patient as an individual.” One second-year resident shared, “they’re really the ones who are specializing in care transitions and are especially knowledgeable about geriatric patient transitions.” Another second-year resident stated,

I didn’t know that pharmacy and social work were so intertwined in the post discharge care, so I thought that was really helpful that there was a chance to hand off to those people as well, in addition to just their provider.

However, some residents raised potential drawbacks with concern that “Sometimes there were too many cooks in the kitchen,” (second-year resident) especially if they did not have prior experience with interdisciplinary care or a framework to guide interactions between professions:

I think a lot of times we just throw a lot of specialties in the same place and think that it’s going to magically change the outcome, but I don’t know what the pharmacist needs from me or what specific questions I can help them with, where I have unique value … or the outpatient nurse or PT or social work. (Second-year resident)

It is important to note that while most residents reported perception of clinical value delivered by TEAM calls, some residents described contradictory attitudes or feelings about the educational value. One second-year resident stated, “I was already aware that it was very important to be very deliberate and clear with our discharge instructions”. Another second-year resident expressed similar sentiments in their position as a senior resident but recognized potential value of participation for interns:

I think it did help my interns a couple times because it highlighted near misses. Maybe they weren’t aware that they had to do it this way for this to be articulated, or maybe they had to re-write the ways that they wanted the medications to be taken. I think it definitely benefited the intern, but I don’t know that it changed the way that I would discharge a patient.

## Discussion

We found that TEAM calls prompted a dawning awareness of residents’ educational blind spots related to transitions of care in elderly patients discharging from the hospital, especially related to residents’ increased appreciation of social factors that can affect health outcomes at discharge, perceptions of their role in patient care within the larger healthcare system, and valuation of a interdisciplinary approach at discharge. Considered together, the three key themes suggest a larger shift from an individualistic to a team-based approach on the part of the resident coordinating patient care after discharge.

Medical student transitions curricula consisting of didactics, experiential learning, and small group discussions have been studied previously [14–16], with one study observing increased awareness of challenges to effective discharge planning, as well as shifting attitude toward a more patient-centered approach [16], similar to our TEAM model in residents.

A limited number of groups have studied how TOC curricula impact resident perceptions of patient care around hospital discharge [7, 8, 17]. One intervention integrated didactics with post-discharge follow up at skilled nursing facilities, nursing homes, and home health nursing visits [7]. In another study, chart reviews of previously hospitalized patients were used as a way for residents to reflect on their practice and understanding of their patients’ needs [8]. A third study involved experiential learning, self-learning by reading an article on TOC [2], facilitated small-group discussions, and writing a self-reflective essay [17]. Several themes consistently emerged in this prior research: the importance of improved TOC planning [7, 8, 17], the importance of effective communication with receiving providers [7, 8, 17], with patients and families [8, 17], and appreciation of social factors that impact safe discharge planning [18]. These themes correlate with those that were evoked by TEAM calls.

Notably, participating in TEAM calls prompted residents to place higher value on other disciplines during transitions of care. Part of this is likely related to the design of the TEAM calls as interdisciplinary in nature. Interestingly, a TOC curriculum in which medical students and pharmacy students worked together to coordinate discharges and post-discharge follow up found a similar increase in appreciation of interdisciplinary care [15]. Because a team approach is necessary to coordinate safe discharge planning, this suggests that future TOC interventions and curricula should incorporate an interdisciplinary focus with inclusion of other disciplines such as pharmacists, social workers and nurse case managers.

Key facilitators for the TEAM calls as an effective intervention included integration into normal clinical workflow for all participants, user-friendly teleconference interface, and a structured script to prompt participation for all inpatient and outpatient team members [18]. These qualities, as well as the interdisciplinary nature of the calls adapted from the original ECHO-CT model, enhanced the sustainability of this model.

### Implications

Overall, residents perceived value of participating in transitions of care TEAM calls that were designed to improve care coordination between inpatient and outpatient settings. Consistent with findings of a systematic review on physician training to provide “high-value, cost-conscious care,” transitions of care curricula should include 1) transmission of specific knowledge with didactics, 2) reflective practice, and 3) a supportive environment [19]. Our TEAM model and other similar curricula consistently demonstrate a positive shift of resident attitude toward transitions of care with increased focus on a team-based approach across disciplines and sites to provide patient care.

### Limitations

Only 11 of 32 residents invited to participate completed interviews and most were senior residents, likely reflective of busy clinical loads. Due to the voluntary nature of this study, our qualitative findings may not be reflective of all the residents who served on the Acute Care of the Elder unit. We continued recruitment until we reached thematic saturation, but we acknowledge that response bias is a limitation of our study. Only residents on the ACE service were interviewed; additional TEAM call participant interviews as well as patient and outpatient provider experiences may shed further light onto the utility of TEAM calls from other perspectives. TEAM calls may not be feasible to implement on inpatient services or clinics that lack adequate support staff or that have inadequate volume of patients cared for by the same inpatient and outpatient care teams.

## Conclusion

TEAM calls provide residents with the opportunity for reflective experiential learning that highlights the importance of a team-based approach, involving multiple disciplines and sites of care, to maximize patient safety and success when discharging from the hospital. Methodical assessment of resident perceptions and attitudes toward TOC curricula can directly inform curricular improvements to optimize the value of resident education in this area. Our findings suggest that blind spots in resident understanding of effective transitions can be identified and addressed by interdisciplinary TEAM calls. Finally, our findings suggest the educational value of experiences with interdisciplinary TOC models may be promoted by reflective debriefs to illuminate previously unrecognized knowledge gaps.

## Supplementary Information

**Additional file 1.** Interview Guide

## Data Availability

The dataset supporting the conclusions of this article are included within the article, in the text as well as the table/supplements. Given the nature of our qualitative interviews, and agreement with our local institutional review board (COMIRB), full interview transcripts/data are securely stored and not publicly available.

## Abbreviations

TOC: Transitions of care
ECHO-CT: Extension for Community Health Outcomes-Care Transitions
SNF: Skilled Nursing Facility
AAIM: Alliance for Academic Internal Medicine
EPA: Entrustable Professional Activities
ACGME: Accreditation Committee of Graduate Medical Education
TEAM: Transitions Expectation and Management
ACE: Acute Care of the Elder
