# Supplementary material for: Resident perspectives on the value of interdisciplinary conference calls for geriatric patients
Source: BMC Med Educ. 2021 Jun 3;21:314. doi: 10.1186/s12909-021-02750-4 (PMC8173720; doi:10.1186/s12909-021-02750-4)
Supplement: Supplementary file 1 — Additional file 1. Interview Guide [file 12909_2021_2750_MOESM1_ESM.docx]

**Additional file 1. Interview Guide**

Interview Guide: Resident Perspectives of Care Transition Calls

- What year of residency are you in?
- Which internal medicine track are you participating in? (Categorical, HTT, Primary Care)

Call content and participants

1. First, I would like to hear from you about the content of the calls. In your opinion, what did you consider to be the main objectives of the call?
2. Was there anything about the contents that stood out to you?
3. Did Social Work or Pharmacy say anything that made an impression on you?
4. What do you think your role in the calls were?
5. Were there unexpected things that were brought up when discussing your patients that you can recall?
6. Are there any benefits that you experienced with patient handoff during the conferences?
7. What about challenges that you encountered with patient handoff during this time?
8. Are you able to describe a specific patient whose care was impacted by the conferences?

Educational value

1. Do you think that experience will impact the way you approach discharge transitions in the future?
2. Is there anything else that you learned from participating in these calls?
3. Do you think that the conferences influenced your assessment of whether or not a patient is ready for discharge?
4. Has participating in the transitions calls changed how you think about your roll in patient care beyond hospital discharge? If so, how?
5. Now I would like to hear in general about the education you have received in residency about care transitions. How did those methods compare to the weekly calls?
6. Did you feel that the main goal of the conferences were for educational purposes or for patient care need?
7. Overall, do you feel that the calls had an impact on patient outcomes after discharge, like length of stay, readmission rates, or patient satisfaction and safety?

Conference call logistics

1. Finally, I would like to hear your thoughts on some logistics of the call. How did the timing of the call impact your daily work flow?
2. What was your overall experience with having the medical record projected and shared with the seniors clinic?
3. How could the format have been improved?
4. Were you able to see the screen projection during the call?
5. Were you able to hear ok on the call?
6. Can you think of others that would have been useful to include in the calls?
7. Are there other way that you believe information exchange between ACE and Seniors could be improved?
8. Did anything else come up while we were talking that you would like to share?
